# Supplementary material for: Reprogramming of Fundamental miRNA and Gene Expression during the Barley-Piriformospora indica Interaction
Source: J Fungi (Basel). 2022 Dec 23;9(1):24. doi: 10.3390/jof9010024 (PMC9865155; doi:10.3390/jof9010024)
Supplement: Supplementary file 1 [file jof-09-00024-s001.zip › jof-2060433-supplementary/supplementary materials/Table S1-S3.pdf]

Table S1. Total reads in mock library

Statu	Total reads	Total bases (bp)	Uniq reads	Uniq bases (bp)
Total Mapped small RNA	7798928	175520380	1070501	24931927
Total Sense Mapped small RNA	6965304	156426315	674937	15723526
Total Antisense Mapped small RNA	833624	19094065	395564	9208401
Total small RNA	8164582	183888022	1243174	28967277

Table S2. Total reads in 3 dai library

Statu	Total reads	Total bases (bp)	Uniq reads	Uniq bases (bp)
Total Mapped small RNA	6418039	148755899	565024	13370541
Total Sense Mapped small RNA	5954841	137904557	355604	8412915
Total Antisense Mapped small RNA	463198	10851342	209420	4957626
Total small RNA	6627084	153594784	666591	15773408

Table S3. Total reads in 7 dai library

Statu	Total reads	Total bases (bp)	Uniq reads	Uniq bases (bp)
Total Mapped small RNA	7136192	169662280	598323	14271709
Total Sense Mapped small RNA	6593838	156800311	368695	8785285
Total Antisense Mapped small RNA	542354	12861969	229628	5486424
Total small RNA	7713820	183511734	739715	17636746
