# Supplementary material for: Reprogramming of Fundamental miRNA and Gene Expression during the Barley-Piriformospora indica Interaction
Source: J Fungi (Basel). 2022 Dec 23;9(1):24. doi: 10.3390/jof9010024 (PMC9865155; doi:10.3390/jof9010024)
Supplement: Supplementary file 1 [file jof-09-00024-s001.zip › jof-2060433-supplementary/supplementary materials/Table S4.pdf]

Table S4. miRNAlibraries quality

Library	Sample	Reads	Bases	Error rate	Q20	Q30	GC content
FRRN202116035-1a	Mock_1	18215949	0.911G	0.01%	99.45%	98.02%	52.51%
FRRN202116035-2a	Mock_2	13103745	0.655G	0.01%	99.51%	98.11%	51.89%
FRRN202116035-3a	Mock_3	14607079	0.730G	0.01%	99.22%	96.95%	52.57%
Hv3DAI_1	Hv3DAI_1	13944721	0.697G	0.01%	99.47%	97.93%	53.05%
Hv3DAI_2	Hv3DAI_2	19256439	0.963G	0.01%	99.42%	97.74%	51.50%
Hv3DAI_3	Hv3DAI_3	13473809	0.674G	0.01%	99.37%	97.47%	51.17%
Hv7DAI_1	Hv7DAI_1	12872083	0.644G	0.01%	99.30%	97.37%	51.68%
Hv7DAI_2	Hv7DAI_2	12244274	0.612G	0.01%	99.47%	97.91%	52.55%
Hv7DAI_3	Hv7DAI_3	17942083	0.897G	0.01%	99.40%	97.73%	53.34%
