# Supplementary material for: Reprogramming of Fundamental miRNA and Gene Expression during the Barley-Piriformospora indica Interaction
Source: J Fungi (Basel). 2022 Dec 23;9(1):24. doi: 10.3390/jof9010024 (PMC9865155; doi:10.3390/jof9010024)
Supplement: Supplementary file 1 [file jof-09-00024-s001.zip › jof-2060433-supplementary/supplementary materials/Table S5.pdf]

Table S5. Primers of target genes and miRNAs used for QPCR.

mRNA Seq-ID	Primer sequence	miRNA	Primer sequence
HORVU7Hr1G012380.5	Forward5'-CGAACAAACAGACGACAAGC-3'<br>Reverse5'-CTGGAGCTGGATGAGG-3'	Novel 1	Forward5'-AGGTGTCCGAAAGAACTTGA-3'<br>Reverse5'-TTGAACTGCTCAACTGAATT-3'
HORVU5Hr1G0055920	Forward5'-AAGGGATCCAACCCAAAATC-3'<br>Reverse5'-GGACGGCTTGGTACTCTCTG-3'		
HORVU3Hr1G076030	Forward5'-CTGGGAGGGTAATCAGCAAA-3'<br>Reverse5'-CGACGAGAGAGAAGGTACGG-3'	mir444b	Forward5'-ACGTCAACGACAGAGTTCGA-3'<br>Reverse5'-TCAAGCTTCTCAACTGAATT-3'
HORVU5Hr1G006020.8	Forward5'-CGCAGTTCCTCTTCAGCTTC-3'<br>Reverse5'-GAAACCAAACAGGCCTGAAA-3'		
HORVU4Hr1G082910	Forward5'-GACTGCTTTCGGCTAGGTTG-3'<br>Reverse5'-TTGACCTTGCCTTTCTGCTT-3'	Novel_22	Forward5'-GACTGTCTTCTCTCACTCGT-3'<br>Reverse5'-GTGAGCACCTCAACTGAATT-3'
HORVU2Hr1G085210	Forward5'-TTGCAGCAATTGTTGAGAGG-3'<br>Reverse5'-CGCTGTGGCAATAGACTTCA-3'		
HORVU5Hr1G054420.4	Forward5'-CTGCGGTGGATCAACTACCT-3'	Novel_40	Forward5'-AAGGTGTCGAAAGAACTTGA-3'<br>Reverse5'-TTGAACTTCTCAACTGAATT-3'

	Reverse5'-TGGTTGTGAGCCTGTGACTC-3'
HORVU7Hr1G088630	Reverse5'-CTCCGGCTACTCACTCCAAG-3'<br>Reverse5'-TGCTGAAGTGGTCTTGATCG-3'
