# Supplementary material for: Reprogramming of Fundamental miRNA and Gene Expression during the Barley-Piriformospora indica Interaction
Source: J Fungi (Basel). 2022 Dec 23;9(1):24. doi: 10.3390/jof9010024 (PMC9865155; doi:10.3390/jof9010024)
Supplement: Supplementary file 1 [file jof-09-00024-s001.zip › jof-2060433-supplementary/supplementary materials/Table S7.pdf]

Table S7. Up-regulated and down regulated targets mRNA in 3 dai vs. Mock

up-regulated targets mRNA				down regulated targets mRNA			
miRNA	log2 FC	Target mRNA	Function	miRNA	log2 FC	Target mRNA	Function
hvu-miR5049a	1.278	HORVU1Hr1G091240	Serine/threonine-protein kinase STY13	hvu-miR444b	-1.4939	HORVU6Hr1G073040	MADS-box transcription factor 57
		HORVU2Hr1G078270	protein LURP-one-related 6	hvu-miR1120	-0.78071	HORVU1Hr1G080480	6-phosphogluconate dehydrogenase
						HORVU3Hr1G039220	pyruvate kinase, cytosolic. isozyme
						HORVU3Hr1G067470	26S proteasome regulatory subunit 6B homolog
		HORVU2Hr1G096290	thioredoxin-like 3-3 isoform X1			HORVU4Hr1G002170	general negative regulator of transcription subunit 3 isoform X4
		HORVU5Hr1G060310	putative transcription factor RL9			HORVU7Hr1G030930	AUGMIN subunit 3
		HORVU6Hr1G025780	protein argonaute 1B	novel_1	-0.90964	HORVU2Hr1G101770	growth-regulating factor 3-like
						HORVU6Hr1G068370	growth-regulating factor 4-like isoform X1
		HORVU5Hr1G112710	leucine rich repeat family expressed			HORVU7Hr1G034610	growth-regulating factor 2-like
		HORVU6Hr1G083120	60S ribosomal protein L35a-1			HORVU6Hr1G081210	growth-regulating factor 1-like
						HORVU7Hr1G008680	growth-regulating factor 5-like isoform X1
		HORVU3Hr1G094730	squamosa promoter-binding-like protein 2			HORVU0Hr1G016610	growth-regulating factor 4-like isoform X2
	1.5432	HORVU6Hr1G028980	cinnamoyI-CoA reductase 1				
novel_22		HORVU6Hr1G	squamosa				

		031450	promoter-binding-like protein 4
		HORVU0Hr1G039170	squamosa promoter-binding-like protein 16
hvu-miR171-3p		HORVU4Hr1G010490	protein MIZU-KUSSEI 1
	1.1618	HORVU4Hr1G087700	scarecrow-like protein 6
		HORVU6Hr1G063650	scarecrow-like protein 27
		HORVU7Hr1G001300	scarecrow-like protein 22
