# Supplementary material for: Reprogramming of Fundamental miRNA and Gene Expression during the Barley-Piriformospora indica Interaction
Source: J Fungi (Basel). 2022 Dec 23;9(1):24. doi: 10.3390/jof9010024 (PMC9865155; doi:10.3390/jof9010024)
Supplement: Supplementary file 1 [file jof-09-00024-s001.zip › jof-2060433-supplementary/supplementary materials/Table S8.pdf]

Table S8. Up-regulated and down regulated targets mRNA in 7 dai vs. Mock

up-regulated targets mRNA				down regulated targets mRNA			
miRNA	log2 FC	Target mRNA	Function	miRNA	log2 FC	Target mRNA	Function
novel_41	1.3484	HORVU4Hr1G056200	vacuolar protein-sorting-associated protein 37 homolog 1-like isoform X3	hvu-miR1120	-1.5238	HORVU1Hr1G080480	6-phosphogluconate dehydrogenase
		HORVU6Hr1G039740	probable serine/threonine-protein kinase SIS8			HORVU3Hr1G039220	pyruvate kinase, cytosolic. isozyme
		HORVU5Hr1G123680	nucleolar protein 12			HORVU3Hr1G067470	26S proteasome regulatory subunit 6B homolog
novel_22	1.5998	HORVU3Hr1G094730	squamosa promoter-binding-like protein 2			HORVU4Hr1G002170	general negative regulator of transcription subunit 3 isoform X4
		HORVU6Hr1G028980	cinnamoyI-CoA reductase 1	hvu-miR444b	-2.1964	HORVU7Hr1G030930	AUGMIN subunit 3
		HORVU6Hr1G031450	squamosa promoter-binding-like protein 4			HORVU6Hr1G073040	MADS-box transcription factor 57
		HORVU0Hr1G039170	squamosa promoter-binding-like protein 16			HORVU1Hr1G000620	transcription initiation factor TFIID subunit 6
				hvu-miR5049d	-1.5549	HORVU3Hr1G002080	rust resistance kinase Lr10-like
						HORVU3Hr1G057530	probable isoprenylcysteine alpha-carbonyl methylesterase ICMEL2 isoform X1
						HORVU4Hr1G025080	probable serine/threonine-protein kinase PBL25
						HORVU7Hr1G021720	probable alpha-1,6-mannosyltransferase MNN10
						HORVU7Hr1G020580	hypothetical protein CFC21_095876
