# Supplementary material for: Reprogramming of Fundamental miRNA and Gene Expression during the Barley-Piriformospora indica Interaction
Source: J Fungi (Basel). 2022 Dec 23;9(1):24. doi: 10.3390/jof9010024 (PMC9865155; doi:10.3390/jof9010024)
Supplement: Supplementary file 1 [file jof-09-00024-s001.zip › jof-2060433-supplementary/supplementary materials/Table S9.pdf]

Table S9. Down regulated targets mRNA in 7 dai vs. 3 dai

miRNA	log2 FC	Target mRNA	Function
hvu-miR171-3p	-1.2508	HORVU4Hr1G010490	protein MIZU-KUSSEI 1
		HORVU4Hr1G087700	scarecrow-like protein 6
		HORVU6Hr1G063650	scarecrow-like protein 27
		HORVU7Hr1G001300	scarecrow-like protein 22
novel_1	-0.70883	HORVU2Hr1G101770	growth-regulating factor 3-like
		HORVU6Hr1G068370	growth-regulating factor 4-like isoform X1
		HORVU7Hr1G034610	growth-regulating factor 2-like
		HORVU6Hr1G081210	growth-regulating factor 1-like
		HORVU7Hr1G008680	growth-regulating factor 5-like isoform X1
		HORVU0Hr1G016610	growth-regulating factor 4-like isoform X2
