# Supplementary figures and images for: Reprogramming of Fundamental miRNA and Gene Expression during the Barley-Piriformospora indica Interaction
Source: J Fungi (Basel). 2022 Dec 23;9(1):24. doi: 10.3390/jof9010024 (PMC9865155; doi:10.3390/jof9010024)

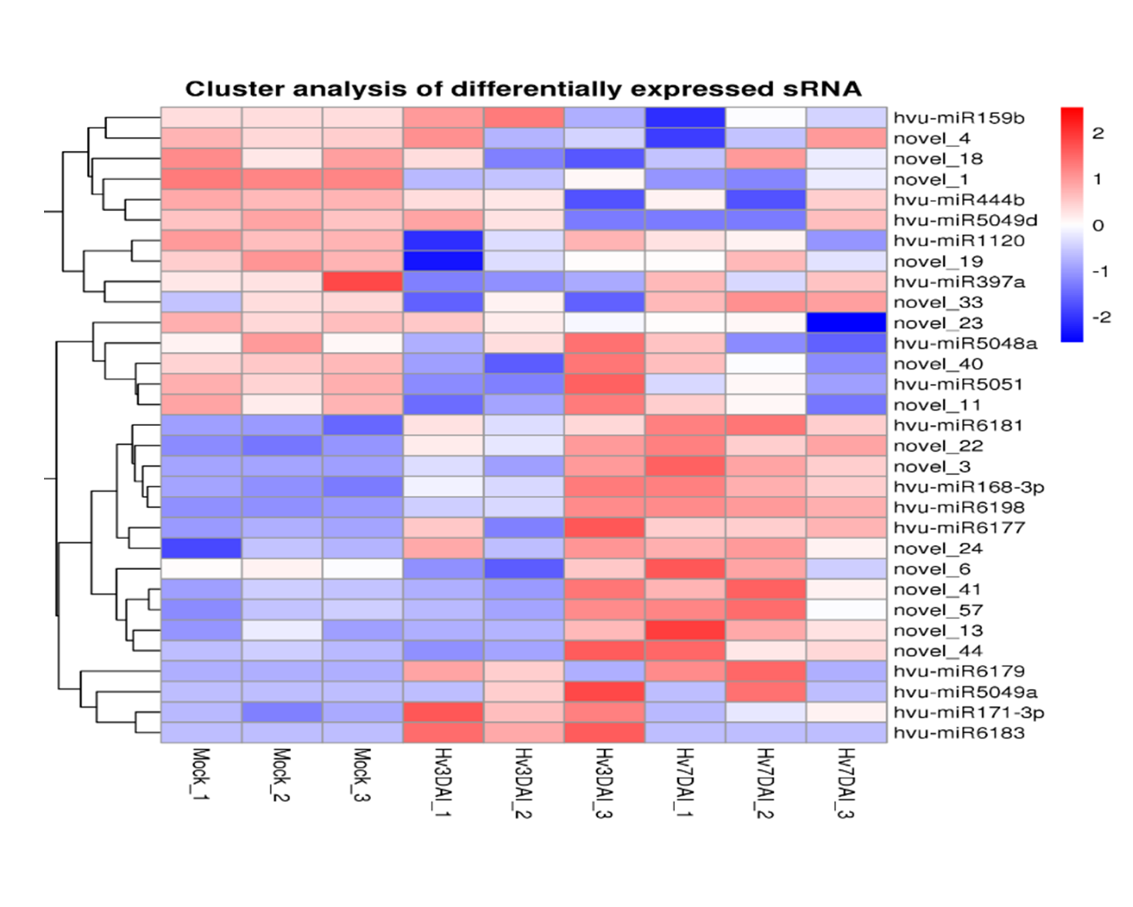

Supplement: Supplementary file 1 [file jof-09-00024-s001.zip › jof-2060433-supplementary/supplementary materials/FIG. S1.tif]
